# Dependence of fullerene aggregation on lipid saturation due to a balance between entropy and enthalpy

**DOI:** 10.1038/s41598-018-37659-4

**Published:** 2019-01-31

**Authors:** Pornkamon Nalakarn, Phansiri Boonnoy, Nililla Nisoh, Mikko Karttunen, Jirasak Wong-ekkabut

**Affiliations:** 1 Department of Physics, Faculty of Science, Kasetsart University, Bangkok, 10900 Thailand; 2 Computational Biomodelling Laboratory for Agricultural Science and Technology (CBLAST), Faculty of Science, Kasetsart University, Bangkok, 10900 Thailand; 3 Thailand Center of Excellence in Physics (ThEP Center), Commission on Higher Education, Bangkok, 10400 Thailand; 4 Department of Chemistry and Department of Applied Mathematics, Western University, 1151 Richmond Street, London, Ontario N6A 5B7 Canada; 5 Specialized Center of Rubber and Polymer Materials for Agriculture and Industry (RPM), Faculty of Science, Kasetsart University, Bangkok, 10900 Thailand

## Abstract

It is well-known that fullerenes aggregate inside lipid membranes and that increasing the concentration may lead to (lethal) membrane rupture. It is not known, however, how aggregation and rupture depend on the lipid type, what physical mechanisms control this behavior and what experimental signatures detect such changes in membranes. In this paper, we attempt to answer these questions with molecular simulations, and we show that aggregation and membrane damage depend critically on the degree of saturation of the lipid acyl chains: unsaturated bonds, or “kinks”, impose a subtle but crucial compartmentalization of the bilayer into core and surface regions leading to three distinct fullerene density maxima. In contrast, when the membrane has only fully saturated lipids, fullerenes prefer to be located close to the surface under the head groups until the concentration becomes too large and the fullerenes begin clustering. No clustering is observed in membranes with unsaturated lipids. The presence of “kinks” reverses the free energy balance; although the overall free energy profiles are similar, entropy is the dominant component in unsaturated bilayers whereas enthalpy controls the fully saturated ones. Fully saturated systems show two unique signatures: 1) membrane thickness behaves non-monotonously while the area per lipid increases monotonously. We propose this as a potential reason for the observations of low fullerene concentrations being effective against bacteria. 2) The fullerene-fullerene radial distribution function (RDF) shows splitting of the second peak indicating the emergence short-range order and the importance of the second-nearest neighbor interactions. Similar second peak splitting has been reported in metal glasses.

## Introduction

Carbon nanoparticles (CNPs), particularly carbon nanotubes and fullerenes, have become very attractive materials from a biomedical point of view due to their unique chemical and physical properties, such as size, hydrophobicity, dimensionality, electronic properties, reproducibility and so on^1–4^. For example, fullerenes are currently being used in cosmetic products, contrast agents, and in drug delivery^2,5–8^. The rapid growth of nanoparticle applications has led to increased production, raising concerns about environmental safety^9–12^. The effects of fullerenes on health and living organisms are a growing concern that has sparked studies of their interactions with biological membranes^13–21^. Such concerns are substantiated in light of damage caused by small particles in silicosis^22^ and suggestions that air pollution from traffic has detrimental effects on fetal growth^23^.

Fullerenes (C60) are nanoparticles that are soluble in various organic solvents^24–26^ and their strong hydrophobicity can be used to assist hydrophilic drugs in translocation across lipid membranes^14,27–29^. On the other hand, in polar solvents such as water, fullerenes form aggregates with the average diameter typically ranging from tens to few hundreds of nanometers^27,30–32^. We have previously shown how fullerenes aggregate in water and how such clusters can passively translocate into lipid membranes^14^. It was further shown that once inside a membrane, the aggregates dissociate and fullerenes become dispersed. It was also observed that the dispersed fullerene molecules stay inside the membrane for simulation times of microseconds with no damage to the bilayer. Free energy analyses have indicated that a dimer is energetically unfavorable due to the distortion of the bilayer structure^33–35^.

The above explanation, however, holds only at low fullerene concentrations^36^. Several experimental and computational studies have shown that fullerene aggregates are harmful to biological membranes^17,19–21,37–39^. However, the experimental results of fullerene aggregation in lipid membrane are still controversial especially since the aggregate sizes are not known^20,40–42^. Importantly, the mechanisms of fullerene aggregation inside the lipid membrane remain poorly understood^14,20,28,37,39,43^. Computer simulations offer a complementary method^44,45^, however, fullerene aggregation may depend on the concentration and the accessible time and length scales set some limitations that necessitate the use of coarse-grained (CG) methods^46^.

In this study, we performed coarse-grained molecular dynamics (CGMD) simulations to investigate the physical mechanisms of fullerene aggregation by varying the concentration of fullerenes in model lipid bilayers. Free energy, enthalpy, and entropy profiles were examined which allowed us to explain the thermodynamics of aggregation. The results show why fullerenes favor dispersion and aggregation in lipid bilayers at low and high concentrations, respectively.

## Methodology

We use CGMD simulations to study the aggregation of fullerenes in lipid membranes at different fullerene concentrations. Saturated and unsaturated lipid membranes are modeled with 1,2-dipalmitoyl-*sn*-glycero-3-phosphocholine (DPPC), 1,2-dioleoyl-*sn*-glycero-3-phosphocholine (DOPC), and 1-palmitoyl-2-oleoyl-*sn*-glycero-3-phosphocholine (POPC) lipids. The simulations were performed by using the GROMACS 4.5.5 package^47^. The Martini force field version 2.1 was used for lipids and water^48^. The coarse grained fullerene molecule (F16) consist of 16 beads and the latest updated fullerene parameters were used to reproduce the atomistic potential of mean force (PMF) profile of transferring a fullerene through a lipid membrane^49^. This fullerene model has been previously validated against experimental solvation free energies and atomistic simulations and it has been used in numerous independent studies with various lipid bilayers (DPPC, POPC, DOPC, DSPC and DUPC) and shown to work well^14,19,21,28,34,39,42,49–53^. The models and atom numberings are shown in Fig. S1. The simulated systems consisted of 512 lipids and 16,000 water CG molecules. The fullerene to lipid ratio was varied between 0 and 0.5. All simulations were carried out under the constant particle number, pressure and temperature (NPT) ensemble. Fullerenes were initially randomly placed in the bulk water phase. Over the course of the simulation they spontaneously came into contact with the membrane, and after a few microseconds fullerenes had passively penetrated inside the bilayer. The simulations were run for 20–50 μs with a 20 fs time step. The last 10 μs were used for analysis and errors were estimated based on the standard deviation. Temperature was controlled using the Parrinello–Donadio–Bussi velocity rescale algorithm^54,55^ with a time constant of 1.0 ps. The Parrinello–Rahman algorithm^56^ with a time constant of 5.0 ps and compressibility of 4.5 × 10^−5^ bar^−1^ was used to keep a constant 1 bar pressure in a semi-isotropic fashion. Periodic boundary conditions were applied in all directions. A cut-off of 1.2 nm was used for the long-range neighbor list. The Lennard-Jones interactions were shifted to zero between 0.9 and 1.2 nm, and the Coulomb potential was shifted to zero between 0 and 1.2 nm. The Visual Molecular Dynamics (VMD) software was used for molecular visualization^57^.

The total simulation time exceeds 500 microseconds. The details of all systems are provided in Table 1, and the data presented in the main text is at the temperature of 298 K. We would like to point out that although the experimentally observed main phase transition of DPPC is at about 314 K^58^, the transition temperature for DPPC within the coarse-grained Martini model has been determined to be 295 ± 5 K^59^. This discrepancy is due to the mapping in the process of coarse-graining, but it is important to notice that although the temperature becomes shifted, the qualitative features remain intact. In addition, it has been shown that modeling lipid systems is a particular strength of the Martini model, see e.g. Refs^46,60–62^. Another related question is how good is the Martini model for describing fullerene aggregation? Monticelli^49^ compared dimerization in different CG models and atomistic models, and showed that while some CG models overestimate dimerization (about 5.6 kJ/mol in water in the case of Chiu *et al*.^63^), the Martini parameterization performs remarkably well, within 2.9 kJ/mol (favorable) in water and 0.5 kJ/mol (unfavorable) in octane. Thus, the current Martini model for fullerenes^49^ provides quite a high resolution in terms of free energy differences. Similarly, Hsu *et al*. used the Martini model to study the interactions of fullerenes and bacterial membranes, and showed very good agreement with both atomistic simulations and experiments^64^. This is different from the case of protein aggregation in membranes where Martini appears to excessively favor dimerization^65^.

**Table 1:** List of the simulated systems. The total simulation time including equilibration exceeds 500 μs.

No.	Fullerene (F16) Concentrations (%)	Molecules					Simulation Time (μs)
		F16	DPPC	DOPC	POPC	Water	
1	0	0	512	—	—	16,000	20
2	5	24	512	—	—	16,000	20
3	10	48	512	—	—	16,000	20
4	20	104	512	—	—	16,000	20
5	30	152	512	—	—	16,000	20
6	40	204	512	—	—	16,000	20
7	50	256	512	—	—	16,000	50
8	0	0	—	512	—	16,000	20
9	5	24	—	512	—	16,000	20
10	10	48	—	512	—	16,000	20
11	20	104	—	512	—	16,000	20
12	30	152	—	512	—	16,000	20
13	40	204	—	512	—	16,000	20
14	50	256	—	512	—	16,000	20
15	0	0	—	—	512	16,000	20
16	5	24	—	—	512	16,000	20
17	10	48	—	—	512	16,000	20
18	20	104	—	—	512	16,000	20
19	30	152	—	—	512	16,000	20
20	40	204	—	—	512	16,000	20
21	50	256	—	—	512	16,000	20

## Results and Discussion

### Membrane dimensions

**Figure 1:**
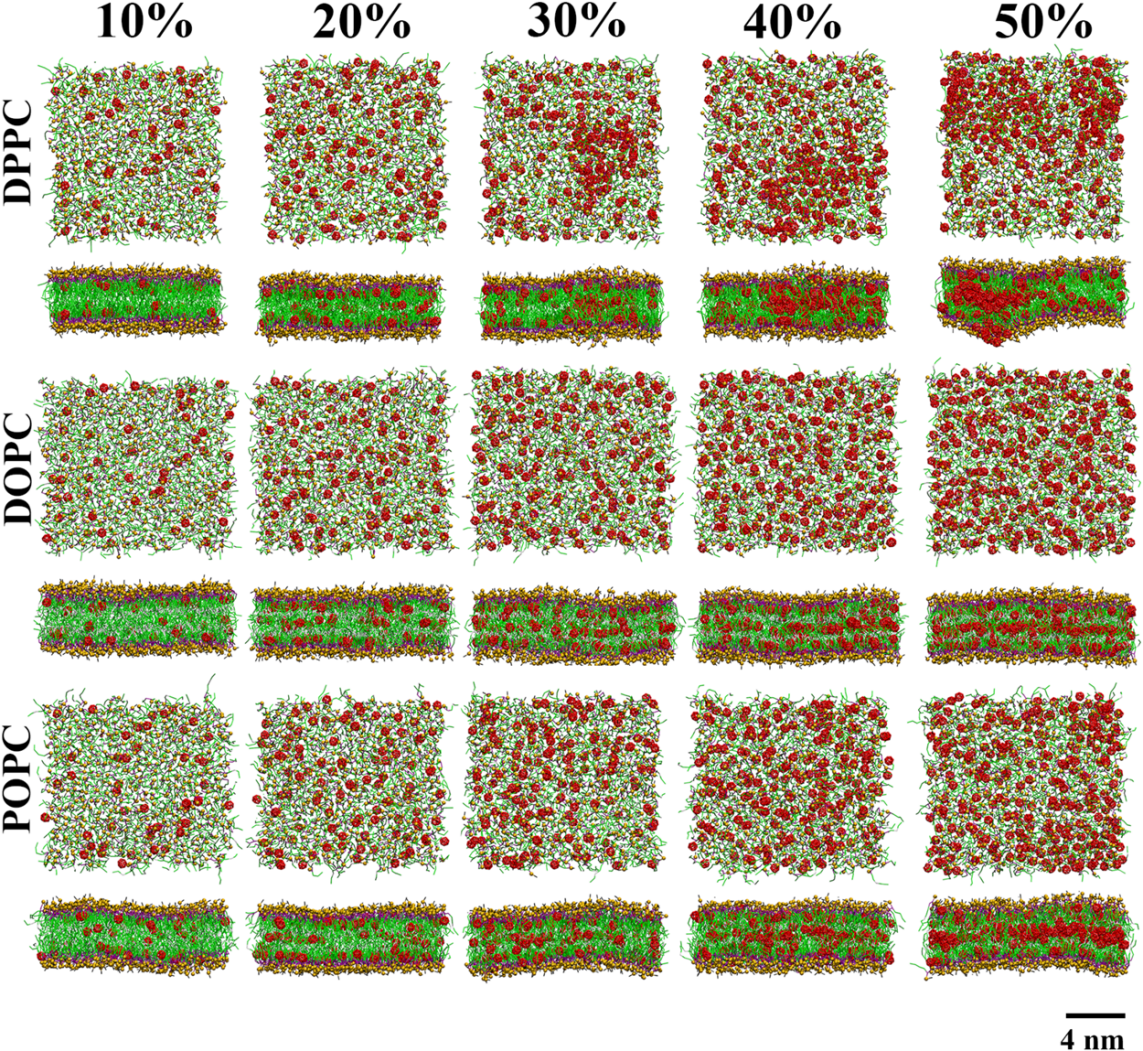
Top and side views at the end of the simulation at different fullerene concentrations in DPPC, DOPC, and POPC lipid bilayer. Water molecules are not shown for clarity. Black, yellow and purple: choline group (NC3), phosphate group (PO4) and glycerol group (GL1 and GL2 bead) of lipid in the Martini model. Green and white: Carbon and double bond bead, respectively. Red spheres: fullerenes molecules.

**Figure 2:**
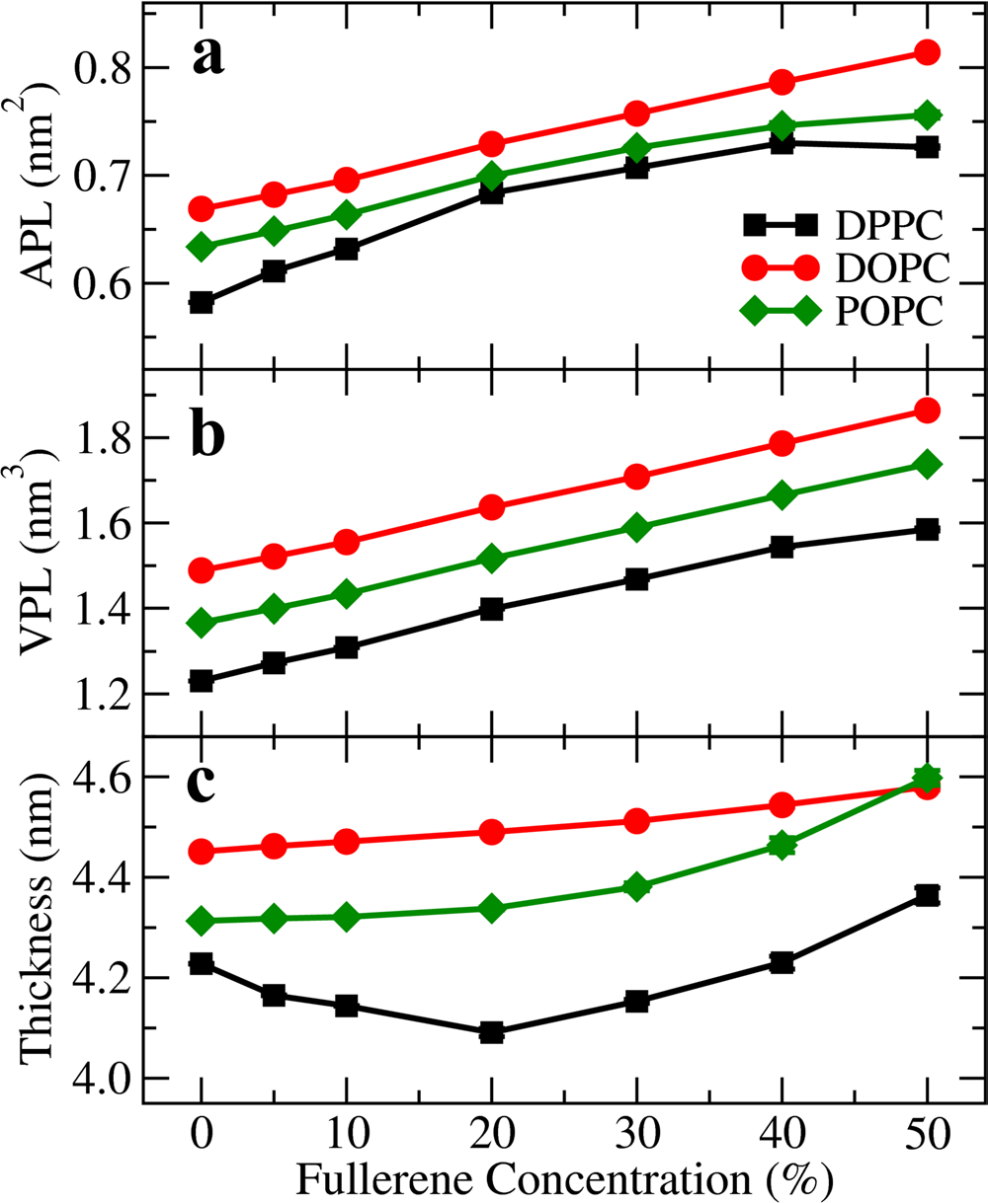
Area per lipid (APL), volume per lipid (VPL) and bilayer thickness at different fullerene concentrations. The error bars are smaller than the size of the symbols (visible under some of the symbols). DPPC, DOPC, and POPC lipid bilayers are represented in black, red and green, respectively.

Figure 1 shows snapshots of structures at the end of the simulations and Fig. S2 shows final structures at additional concentrations for completeness. As the figures show, aggregation was observed to depend on fullerene concentration in accord with previous studies^28,34^. Fig. 2a,b show that both the area (Fig. 2a) and volume per lipid (Fig. 2b) of DPPC, DOPC and POPC bilayers increase with increasing fullerene concentration. The area per lipid has been studied before and our results are in agreement with previous studies^14,21,28,66^. We also analyzed membrane thickness, Fig. 2c. The thicknesses of both the DOPC and POPC bilayers increase monotonously with increasing fullerene concentration in agreement with previous studies^14,66^.

In contrast, however, the thickness of the DPPC bilayer shows non-monotonous behavior as it decreases at fullerene concentrations below 20% where it reaches its minimum, after which the thickness starts to increase. This non-monotonous change in thickness appears to be characteristic of fully saturated (here DPPC) bilayers only. We return to the origin of this below in the discussion regarding the free energy, but the top views in Fig. 1 show (see the difference between 20% and 30%) that qualitatively, this is linked to the clustering of fullerenes on the membrane surface and their subsequent entry to the membrane interior. A weak non-monotonous behavior has also been reported (albeit not discussed) by Gupta and Rai in simulations of *stratum corneum* (the model consisted of a mixture of free fatty acids, long ceramides and cholesterol) with fullerenes^21^.

**Figure 3:**
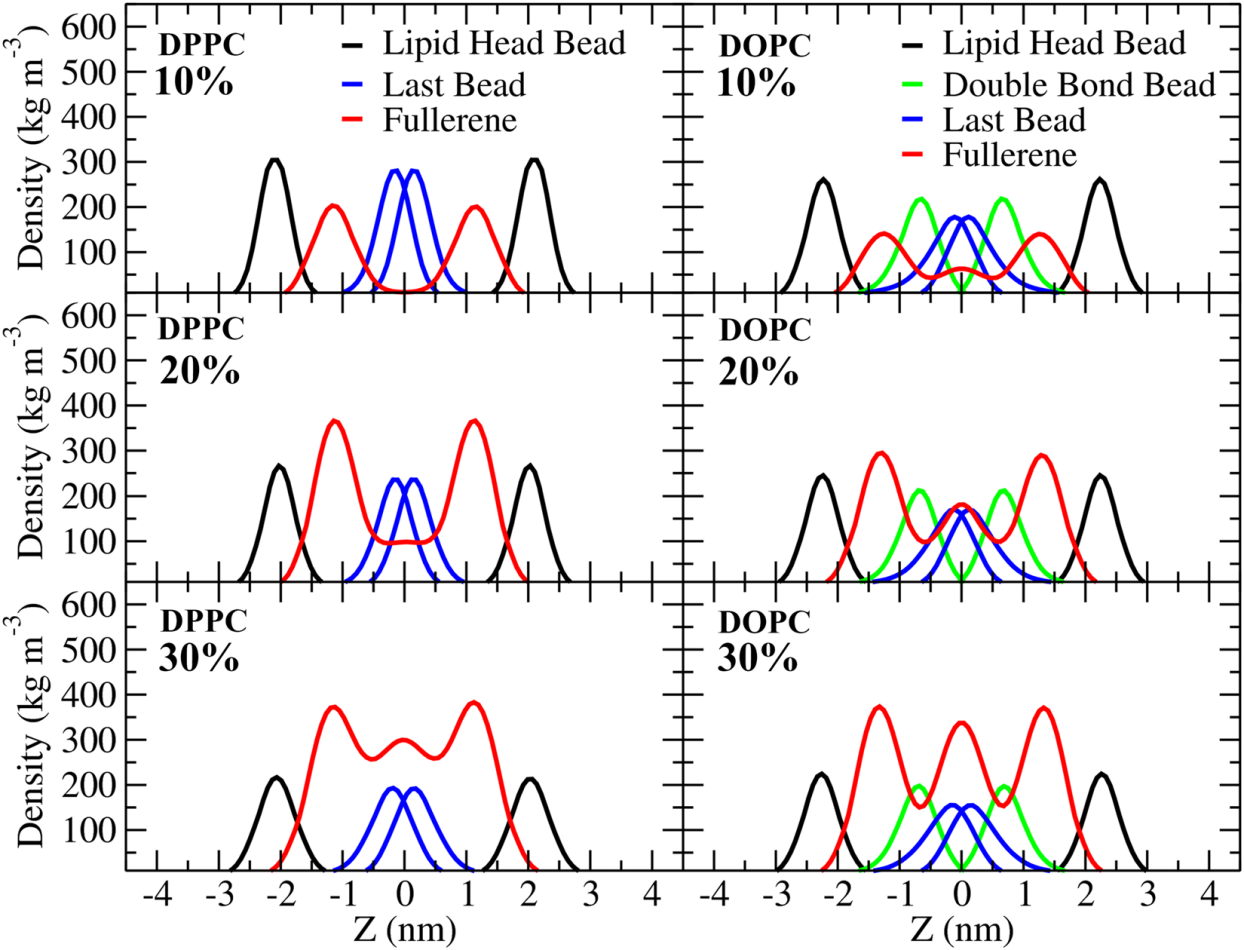
Mass density profiles of the lipid components and fullerene along the bilayer normal (z-axis) at 10%, 20%, and 30% of fullerene concentration in DPPC and DOPC bilayers. Note that lipid head bead, double bond bead and the last bead are PO4, D3B and C4B(DPPC)/C5B(DOPC), repectively.

As is clear from Fig. 1, systems with double bonds (POPC and DOPC) have qualitatively different behavior from the fully saturated DPPC system: instead of clustering, fullerenes organize themselves in layers. To characterize this behavior, we measured the component-wise density profiles across the bilayers, Fig. 3 (see in Figs. S3, S4). As the figure shows, at concentrations up to 20%, fullerenes stay close to the surface in DPPC whereas in DOPC fullerenes layer into three different regions beginning at concentrations of 5% and becoming pronounced at 20%, subsequently developing in a systematic fashion. This is also evident in the side views shown in Fig. 1. This layering is induced by the double bonds whose density is also shown in Fig. 3. Although the area and volume per lipid are higher in the DOPC bilayer, the double bonds present a subtle constriction thus dividing the membrane interior into three regions. In the case of DPPC the area per lipid is smaller and at increasing concentrations it appears that after saturating the region close to the head groups, the presence of a larger local distortion becomes more likely. Note that the largest bilayer deformation was observed at 50% concentration of fullerene in DPPC bilayer caused by the accumulation of fullerenes inside the bilayer. To examine changes in elastic properties, we computed the isothermal area compressibility in all the cases (Fig. S5). At zero fullerene concentration the values are in excellent agreement with those obtained by Daily *et al*.^67^. For concentrations up to 20%, DOPC and POPC show qualitatively similar behavior while compressibility for DPPC increases rapidly and exceeds that of both the POPC and DOPC systems at about 10%. This rapid increase is correlated with decreasing thickness as reported in Fig. 2c. At higher concentrations (30% and above), the error bars become large due to lack of data (correlation time increases). Quantitative analysis of fullerene clusters will be shown in the next section.

### Fullerene aggregation

**Figure 4:**
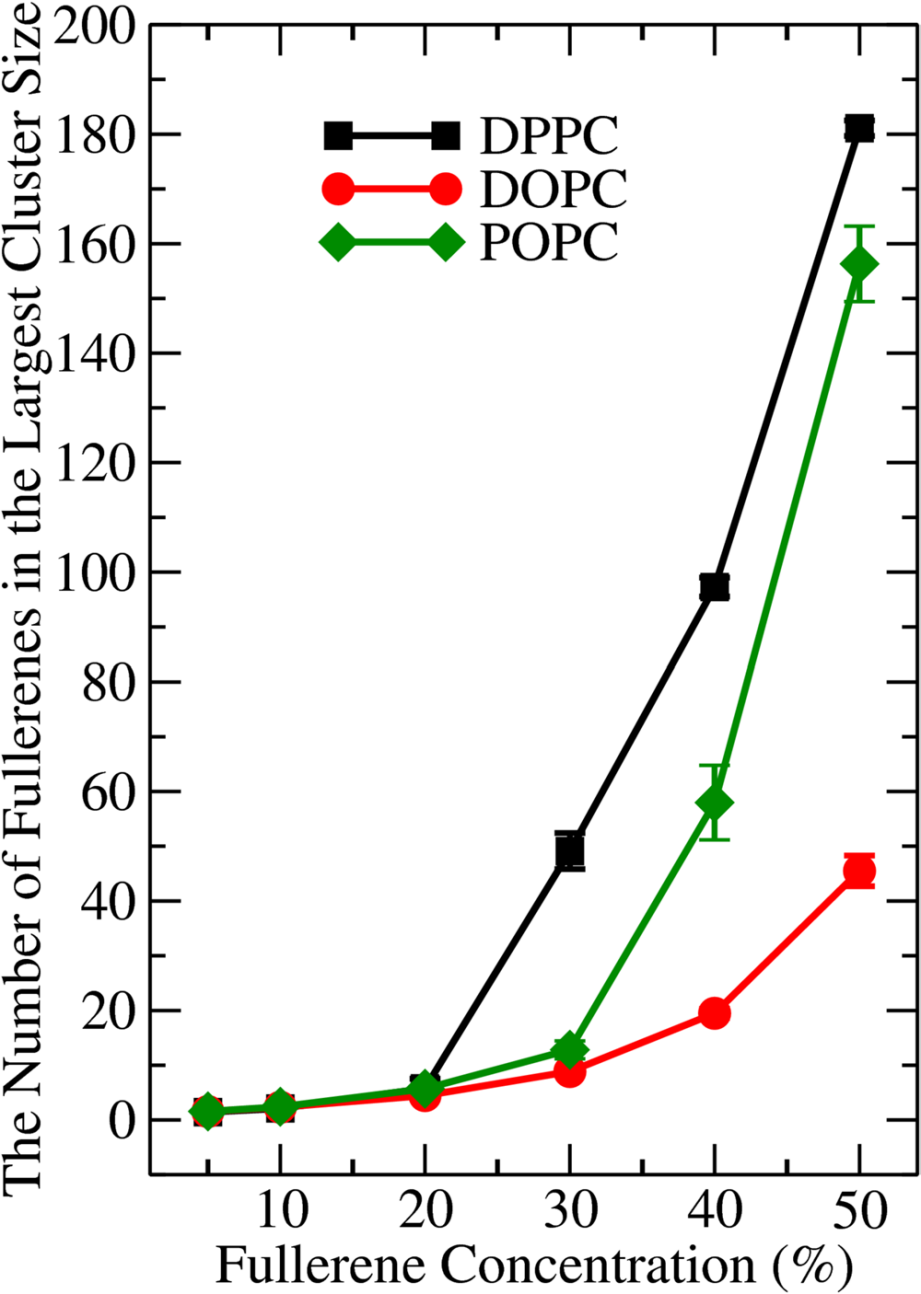
The average number of fullerenes in the largest cluster size as a function of fullerene concentration in DPPC, DOPC, and POPC bilayers.

To study the aggregation of fullerenes in lipid bilayer, we used previously validated software from Barnoud *et al*.^34^. Two fullerene molecules are considered to be clustered when their centers of masses are closer than 1.30 nm^19,34^. Fig. 4 shows that the largest cluster increases with increasing fullerene concentration and Fig. S6 shows the time dependence of cluster formation in two representative cases. In the case of DPPC, the onset of rapid cluster growth starts around 20%, correlating well with the minimum in the non-monotonous behavior of bilayer thickness in Fig. 2c. Aggregation in DPPC and DOPC (POPC is similar to DOPC) is qualitatively different from each other. As Fig. 4 shows, the cluster sizes are significantly larger in the case of DPPC, whereas in DOPC and POPC, only small clusters are observed; the percentage of fullerenes belonging to the largest cluster is up to ~70% in DPPC (the largest cluster did not span all of the fullerenes) and only 20% in DOPC bilayers (Fig. S7).

**Figure 5:**
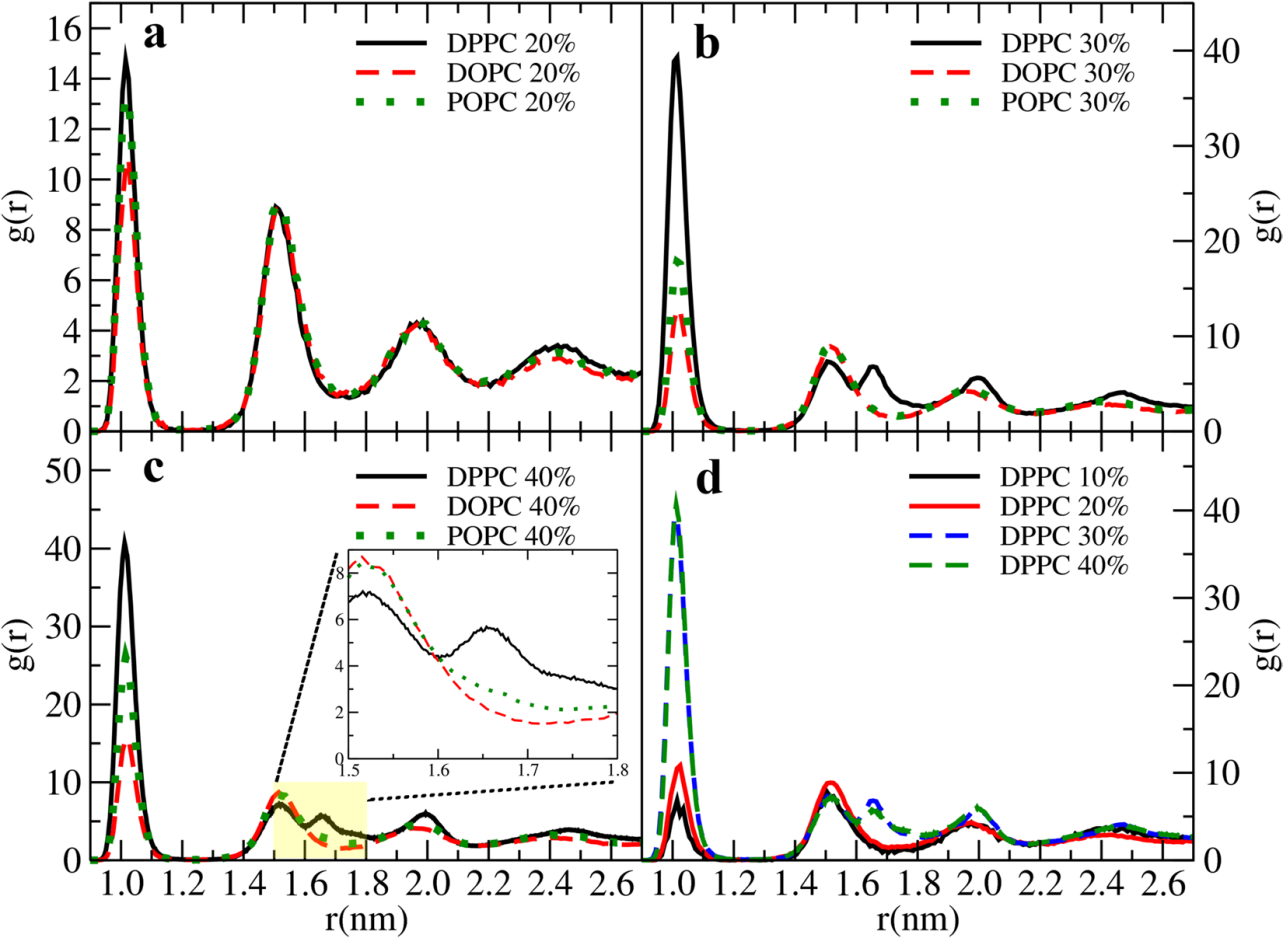
Radial distribution functions of fullerenes inside lipid bilayer at different fullerenne concentrations. The inset in c shows the detailed structure between 1.5 and 1.8 nm (the yellow box) in particular how the second peak of the POPC RDF has started to broaden.

To further analyze aggregation, we determined the center-of-mass positions for fullerenes as a function of time in each case and computed the (unnormalized due to the thin film geometry) radial pair distribution function (RDF) for them. In the case of the unsaturated DOPC matrix, the RDF is liquid-like (Fig. 5). This is drastically different in the DPPC matrix (Fig. 5d): While below 20% the RDFs are liquid-like, the second peak of the RDF has fully split at 30%. In the case of POPC, the RDF displays a characteristic broadened shoulder at 40% (the inset in Fig. 5c shows the onset). DOPC with double bonds in both of its tails shows no signs of such behavior even at 40%. These results together with the density profiles in Fig. 3 show that the peak splitting is due to lipid saturation (double bond position is indicated in the figure).

Peak splitting is often observed in metallic glasses and liquids. The second peak is due to, in general, second nearest neighbors. As the analysis of Ding *et al*. shows^68^, splitting arises when more than two atoms share parts of the second-nearest neighbor shell. In terms of their three dimensional Voronoi cells, splitting arises when the second shells share more than a single vertex (a single vertex equals to one atom and leads to liquid-like behavior), that is, they have to share either an edge or a face of their Voronoi cell indicating short range order^68^. This order is due to cluster formation in the DPPC system with fully saturated chains.

### Free energy

**Figure 6:**
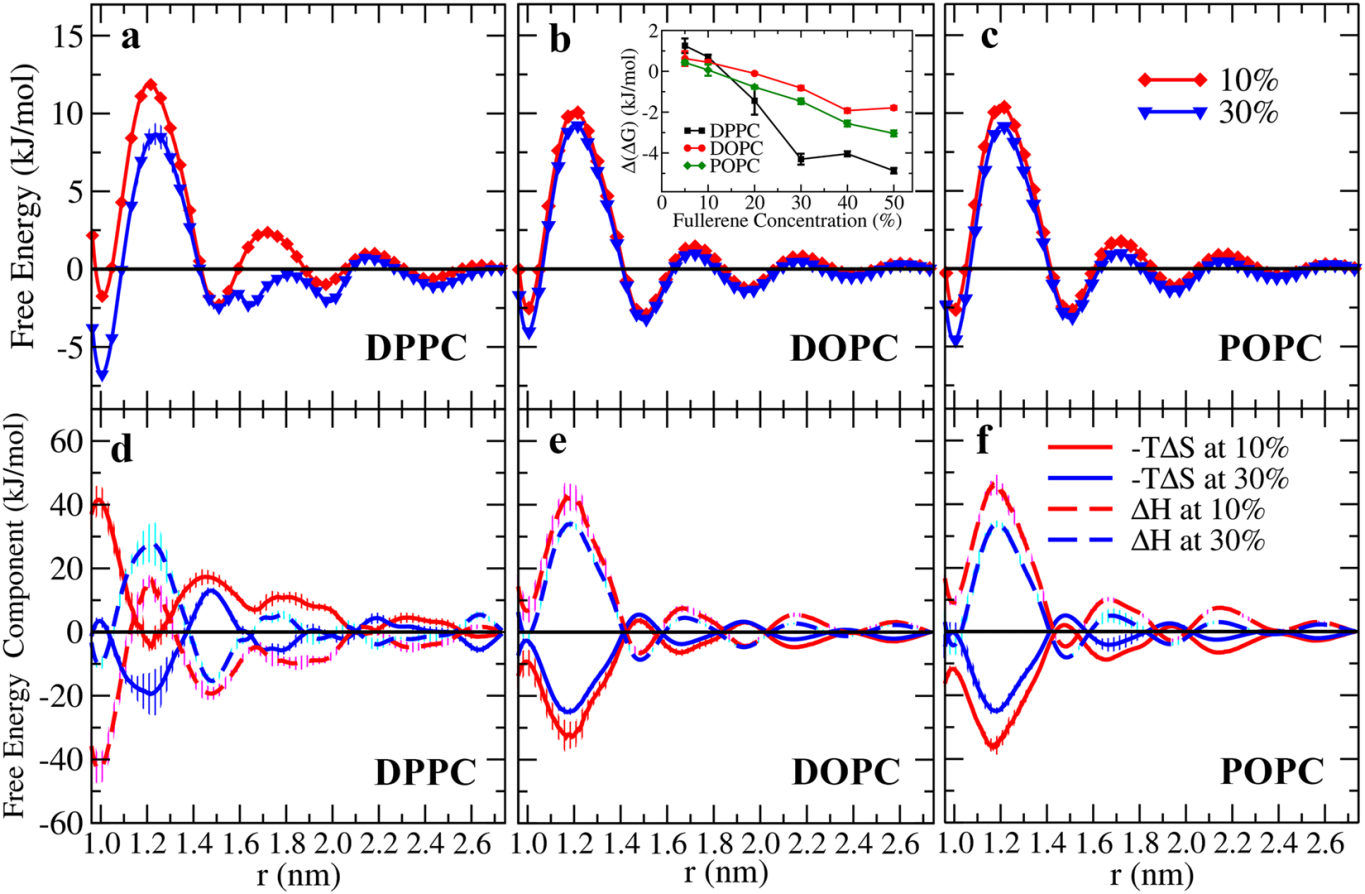
(**a**–**c**) Free energy at 10% and 30% fullerene concentrations, other concentrations are shown in Figs. S8 and S9. The first and second minima of free energy are at the distances between fullerenes of 1.0 and 1.5 nm, respectively. (**d**–**f**) Entropic (solid lines) and enthalpic (dashed lines) components of the free energy calculated at 10% and 30%. The entropic component of free energy is -T∆S. Inset shows the free energy difference between the first and second minima. Thermal energy is about 2.5 kJ/mol. The standard deviation was used for error estimates.

**Figure 7:**
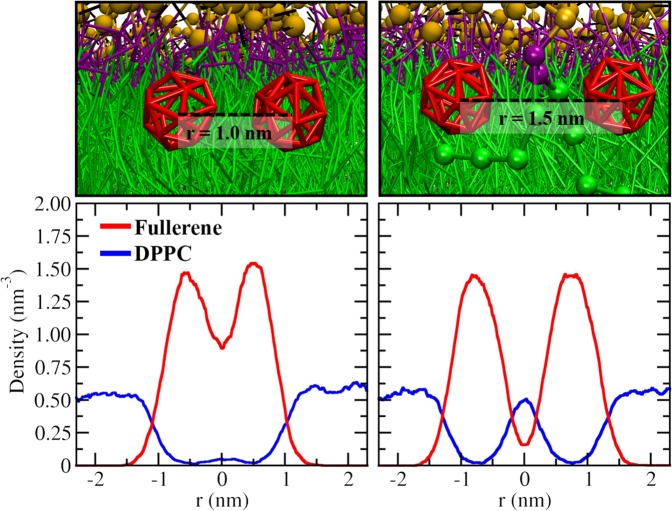
The density of fullerenes (red) and lipids (blue) along the vector connecting two fullerenes (in-plane) at displacements of 1.0 and 1.5 nm.

Figure 6 shows the free energy profiles and the decomposition of free energy into its entropic and enthalpic components. The free energy profiles were calculated using

1 $${\rm{\Delta }}G(r)=-\,{k}_{B}{\rm{T}}\,\mathrm{ln}\,[{\rm{g}}(r)]$$

where ${k}_{B}$ is the Boltzmann constant, $T\,\,$is the absolute temperature, and ${\rm{g}}(r)$ is the radial distribution function with *r* being the distance between two fullerenes^69^. Figs. 6 and S8 show that the first and second minima of the free energy are always located at 1.0 and 1.5 nm, respectively (Fig. 6a–c); at 1.0 nm two neighboring fullerenes are in contact. The positions of first and second minima are in agreement with the work of Barnoud *et al*. who studied the interactions of fullerenes and POPC membranes and found dispersion of fullerenes up to concentrations of 18% (the highest concentration in that study)^34^. Figs. 6a–c also show that at low concentrations the global minimum is at 1.5 nm (the second minimum), meaning that fullerenes prefer to be dispersed as monomers. The origin of the second minimum at 1.5 nm is qualitatively similar to what was observed by Cao *et al*.^70^ who studied fullerenes in ethanol solutions. They observed a second (albeit shallow) minimum and traced that to be due to the fullerene-fullerene distance at which one of ethanol’s functional groups is able to become inserted between a pair of fullerenes. Here, 1.5 nm is the distance at which a lipid bead can be inserted between two fullerenes. This is demonstrated in Fig. 7 which shows the in-plane density between fullerenes at separations of 1.0 nm and 1.5 nm. The free energy decomposition in Fig. 6 also shows that independent of the system and fullerene concentration, the enthalpic component always shows a minimum at that distance. The above suggests that fullerenes do not damage biological membranes at low concentrations via aggregation and consequent buckling of the membrane. We would like to point out, however, that thinning of the saturated DPPC membrane with simultaneous increase in the area per lipid, may expose the membrane to, for example, leakage. This is consistent with the observations of Lyon *et al*.^71^ who reported that fullerenes have a strong antimicrobial effect (against *B. subtilis*) at low concentrations.

At around 20%, the first minimum at 1.0 nm becomes the global one indicating that aggregation becomes dominant. Furthermore, as the concentration increases from 20% to 30%, in the case of DPPC the second maximum breaks into two smaller maxima separated by a shallow local minimum. This is consistent with the non-monotonicity observed in membrane thickness (Fig. 2c) and the growth of clusters (Fig. 4). No such splitting occurs in the case of DOPC. To study the effect of system size on free energy profiles, we simulated larger systems consisting of 2048 DPPC lipids with 10% and 30% fullerene concentrations. The results did not show any significant differences (Fig. S9).

To understand this better, following MacCallum *et al*., the free energy of lipids bilayer was decomposed into its entropic and enthalpic components as^72^.

2 $$-\,{\rm{T}}{\rm{\Delta }}{\rm{S}}={\rm{T}}\frac{{\rm{dG}}}{{\rm{dT}}}\approx \frac{{\rm{T}}}{2{\rm{\Delta }}{\rm{T}}}({\rm{G}}({\rm{T}}+{\rm{\Delta }}{\rm{T}})-{\rm{G}}({\rm{T}}-{\rm{\Delta }}{\rm{T}}))$$

3 $${\rm{\Delta }}{\rm{H}}={\rm{\Delta }}{\rm{G}}+{\rm{T}}{\rm{\Delta }}{\rm{S}}\,$$

Additional simulations were performed with varying temperatures of 283 and 313 K in order to obtain a reliable estimate for the entropic component. The results are shown in Fig. 6d–f. First, Fig. 6a shows that as the fullerene concentration in a DPPC bilayer increases from 10 to 30%, the dimerization free energy (*r* = 1.0 nm) decreases from −1.75 to −6.76 kJ/mol. Fig. 6d shows that this is due to the entropic component decreasing faster than the enthalpic component increases. This is in contrast to DOPC where the entropic component dominates; this is similar to POPC bilayers studied by Barnoud *et al*.^34^ and also verified here (data not shown). Thus, the above results clearly show that the aggregation behavior is controlled by the balance between entropy and enthalpy, and that this depends on whether the acyl chains are saturated or not. Meanwhile the free energy of the second minimum decreases only slightly (~0.15 kJ/mol) and the changes in the enthalpic and entropic components compensate each other (thermal energy is about 2.5 kJ/mol). The inset in Fig. 6b shows the free energy difference between the first and the second minima as a function of concentration. The difference increases faster for the fully saturated DPPC. Importantly, the thermal energy corresponds to about 2.5 kJ/mol indicating (together with the fact that the enthalpic component dominates the free energy) that in DPPC, aggregation is preferred at concentrations above 20%. In DOPC, however, the thermal energy is enough to disperse the fullerenes even at the highest concentrations.

### Diffusion

**Figure 8:**
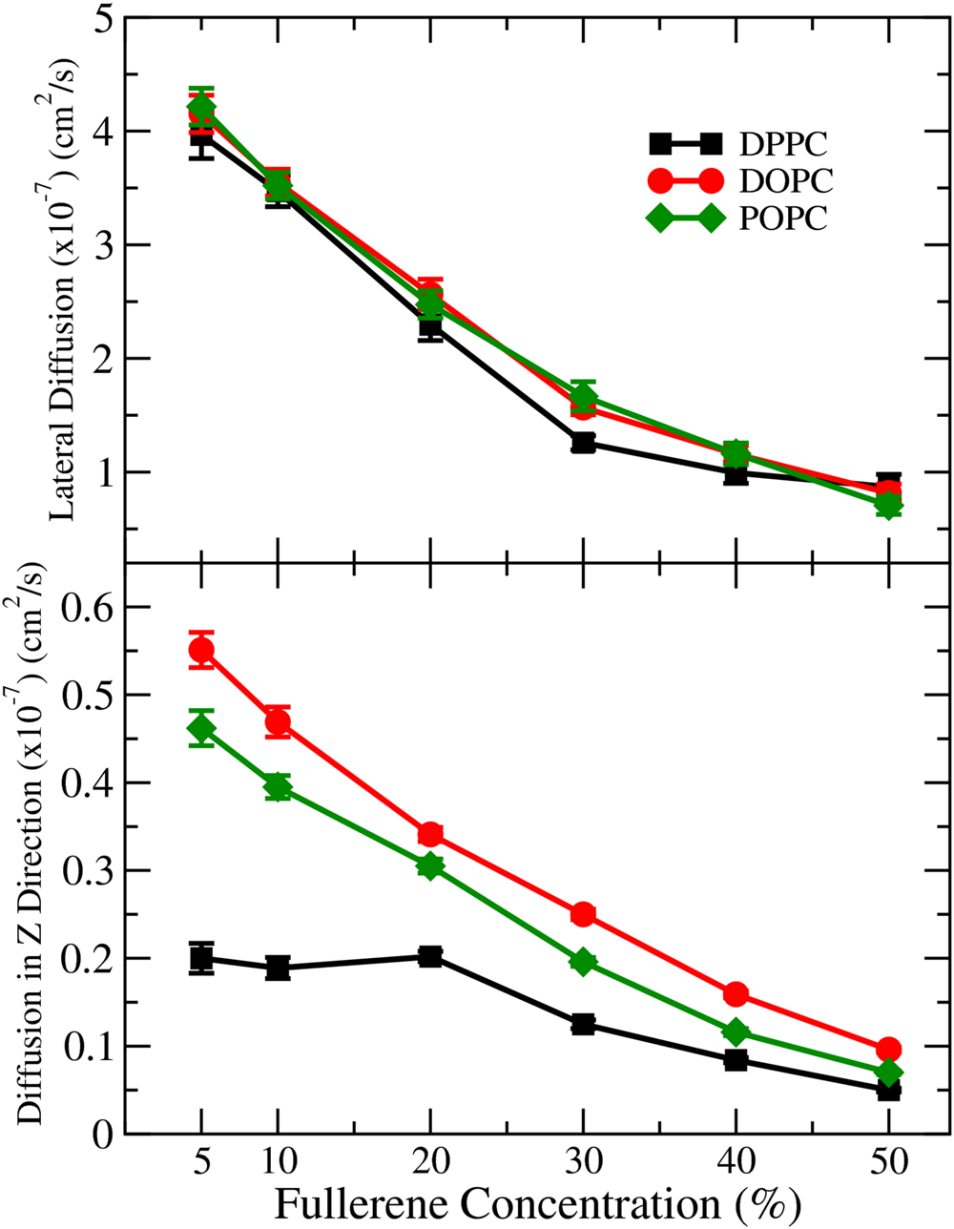
Diffusion coefficients of fullerenes in different lipid bilayers.

Finally, we also computed fullerene diffusion coefficients using mean square displacements both laterally and in the direction normal to the membrane surface. The data is shown in Fig. 8. Slower diffusion was observed at higher fullerene concentrations in agreement with Sastre *et al*.^39^. Perhaps surprisingly, the differences in lateral fullerene diffusion in the three cases are very small. Diffusion in the direction normal to membrane surface, however, shows clear differences: diffusion in DOPC and POPC is very similar, but up to 20% concentration the DPPC system behaves differently with the diffusion coefficient being practically independent of fullerene concentration. The plausible origin of this appears to be explained by Fig. 2: while the area per lipid increases for all systems, thickness of the DPPC systems decreases up to 20% fullerene concentration.

## Conclusions

We have investigated the aggregation of fullerenes in lipid bilayers using coarse-grained molecular dynamics simulations over the total time of more than 500 microseconds. We focused on two lipid matrices: 1) DOPC in which both of the acyl chains have a double bond and 2) the fully saturated DPPC. Third matrix, POPC, was used as a control. We decomposed the free energy into its entropic and enthalpic components at different fullerene concentrations. We show, to our knowledge, for the first time that in the fully saturated bilayers fullerenes are controlled by the enthalpic term which favors aggregation. This is in contrast to DOPC (and POPC) in which the entropy dominates and favors dispersion. This is manifested in the layer-like ordering of fullerenes inside the DOPC bilayer and, the emergence of splitting of the second peak in the fullerene-fullerene center-of-mass pair correlation function in the saturated DPPC matrix as the fullerene concentration increases.

These results have several consequences: First, several experimental studies have shown that fullerenes have antibacterial effects. It has been suggested that this is due to the increased area per lipid. However, as our results and those of others show, the area per lipid increases with increasing fullerene concentration independent of the lipid type. Here we have shown, however, that membranes consisting of fully saturated lipids become thinner at small and moderate fullerene concentrations yet their area per lipid increases monotonously with increasing fullerene concentration. This is consistent and can explain the observations of Lyon *et al*.^71^ who reported that the antibacterial effects against *B. subtilis* were the strongest at small fullerene concentrations. Their data also shows (albeit not discussed) that the area per volume ratio changed non-monotonically. Here, we focused on the interactions of fullerenes with pure bilayers. One of the interesting questions is what happens when membranes have varying compositions. Sastre *et al*.^39^ studied cholesterol-containing membranes (PC-Chol-fullerene) and reported that cholesterols and fullerenes do not prefer to be in contact with each other and that the presence of fullerenes may suppress the inter-leaflet flip-flop rate. They also simulated raft-like membranes and observed that fullerenes prefer the more disordered matrix. There are several open questions including how the asymmetric distribution of cholesterol and different lipid types in real cellular membranes influence the partitioning of fullerenes (and nanoparticles in general) between the leaflets, and how the local variations in the available free volume (e.g., due to presence of cholesterol) influence the distribution. Many of the issues remain unresolved yet they have very important consequences both for applications as well as real biological systems as discussed, e.g., by Chen and Bothun^73^.

These results also open the questions of how the balance between entropy and enthalpy depends on the number or/and location of double bonds, and if by modulating the positions allows for localization of fullerenes in some particular region of a membrane. Finally, we would also like to emphasize that our predictions are experimentally testable using model bilayer systems.

## Supplementary information

Supporting Information

## References

[1] Prato, M. *Fullerenes and Related Structures. In Topics In Current Chemistry*. Vol. 199 (Springer-Verlag 1999).

[2] Bakry R (2007). Medicinal applications of fullerenes. Int. J. Nanomed..

[3] Goodarzi S, Da Ros T, Conde J, Sefat F, Mozafari M (2017). Fullerene: biomedical engineers get to revisit an old friend. Mater. Today.

[4] Castro E, Garcia AH, Zavala G, Echegoyen L (2017). Fullerenes in biology and medicine. J. Mater. Chem. B.

[5] Lalwani G, Sitharaman B (2013). Multifunctional fullerene-and metallofullerene-based nanobiomaterials. Nano LIFE.

[6] Shu CY (2008). Conjugation of a water-soluble gadolinium endohedral fulleride with an antibody as a magnetic resonance imaging contrast agent. Bioconjugate Chem..

[7] Jensen AW, Wilson SR, Schuster DI (1996). Biological applications of fullerenes. Bioorganic Med..

[8] Leszek J (2017). Nanotechnology for Alzheimer Disease. Curr. Alzheimer Res..

[9] Colvin VL (2003). The potential environmental impact of engineered nanomaterials. Nat. Biotechnol..

[10] Lujan H, Sayes CM (2017). Cytotoxicological pathways induced after nanoparticle exposure: studies of oxidative stress at the ‘nano-bio’ interface. Toxicol. Res..

[11] Sireesha M, Babu VJ, Ramakrishna S (2017). Functionalized carbon nanotubes in bio-world: applications, limitations and future directions. Mater. Sci. Eng. B.

[12] Monserrat JM (2017). Interference of single walled carbon nanotubes (SWCNT) in the measurement of lipid peroxidation in aquatic organisms through TBARS assay. Ecotoxicol. Environ. Saf..

[13] Sayes CM (2004). The differential cytotoxicity of water-soluble fullerenes. Nano Lett..

[14] Wong-Ekkabut J (2008). Computer simulation study of fullerene translocation through lipid membranes. Nat. Nanotechnol..

[15] Sayes CM (2005). Nano-C-60 cytotoxicity is due to lipid peroxidation. Biomaterials.

[16] Ray PC, Yu HT, Fu PP (2009). Toxicity and environmental risks of nanomaterials: challenges and future needs. J. Environ. Sci. Health C.

[17] Zupanc J (2012). Experimental evidence for the interaction of C-60 fullerene with lipid vesicle membranes. Carbon.

[18] Santos SM (2014). Interaction of fullerene nanoparticles with biomembranes: from the partition in lipid membranes to effects on mitochondrial bioenergetics. Toxicol. Sci..

[19] Nisoh N, Karttunen M, Monticelli L, Wong-ekkabut J (2015). Lipid monolayer disruption caused by aggregated carbon nanoparticles. RSC Adv..

[20] Russ KA (2016). C-60 fullerene localization and membrane interactions in RAW 264.7 immortalized mouse macrophages. Nanoscale.

[21] Gupta R, Rai B (2017). Molecular dynamics simulation study of translocation of fullerene C-60 through skin bilayer: effect of concentration on barrier properties. Nanoscale.

[22] Leung CC, Yu ITS, Chen W (2012). Silicosis. Lancet.

[23] Smith RB (2017). Impact of London’s road traffic air and noise pollution on birth weight: retrospective population based cohort study. BMJ.

[24] Ruoff RS, Tse DS, Malhotra R, Lorents DC (1993). Solubility of fullerene (C60) in a variety of solvents. J. Phys. Chem..

[25] Peerless JS, Bowers GH, Kwansa AL, Yingling YG (2015). Fullerenes in aromatic solvents: correlation between solvation-shell structure, solvate formation, and solubility. J. Phys. Chem. B.

[26] Boucher D, Howell J (2016). Solubility characteristics of PCBM and C60. J. Phys. Chem. B.

[27] Fortner JD (2005). C-60 in water: nanocrystal formation and microbial response. Environ. Sci. Technol..

[28] Xie LQ (2017). Computer simulations of the interaction of fullerene clusters with lipid membranes. Mol. Simul..

[29] Behzadi S (2017). Cellular uptake of nanoparticles: journey inside the cell. Chem. Soc. Rev..

[30] Ha Y, Katz LE, Liljestrand HM (2015). Distribution of fullerene nanoparticles between water and solid supported lipid membranes: thermodynamics and effects of membrane composition on distribution. Environ. Sci. Technol..

[31] Lyon DY, Adams LK, Falkner JC, Alvarez PJJ (2006). Antibacterial activity of fullerene water suspensions: effects of preparation method and particle size. Environ. Sci. Technol..

[32] Andrievsky G, Klochkov V, Derevyanchenko L (2005). Is the C-60 fullerene molecule toxic?!. Fuller. Nanotub. Car. N..

[33] Li LW, Davande H, Bedrov D, Smith GD (2007). A molecular dynamics simulation study of C-60 fullerenes inside a dimyristoylphosphatidylcholine lipid bilayer. J. Phys. Chem. B.

[34] Barnoud J, Rossi G, Monticelli L (2014). Lipid membranes as solvents for carbon nanoparticles. Phys. Rev. Lett..

[35] Barnoud J, Rossi G, Marrink SJ, Monticelli L (2014). Hydrophobic compounds reshape membrane domains. PLOS Comput. Biol..

[36] Zhou J, Liang D, Contera S (2015). Effect of intra-membrane C60 fullerenes on the modulus of elasticity and the mechanical resistance of gel and fluid lipid bilayers. Nanoscale.

[37] Salonen E (2008). Real-time translocation of fullerene reveals cell contraction. Small.

[38] Van der Paal J, Neyts EC, Verlackt CCW, Bogaerts A (2016). Effect of lipid peroxidation on membrane permeability of cancer and normal cells subjected to oxidative stress. Chem. Sci..

[39] Sastre J, Mannelli I, Reigada R (2017). Effects of fullerene on lipid bilayers displaying different liquid ordering: a coarse-grained molecular dynamics study. Biochim. Biophys. Acta.

[40] Hou WC, Moghadam BY, Westerhoff P, Posner JD (2011). Distribution of fullerene nanomaterials between water and model biological membranes. Langmuir.

[41] Ikeda A (2012). Advantages and potential of lipid-membrane-incorporating fullerenes prepared by the fullerene-exchange method. Chem. Asian J..

[42] Rossi G, Barnoud J, Monticelli L (2013). Partitioning and solubility of C-60 fullerene in lipid membranes. Phys. Scr..

[43] Tian WD, Chen K, Ma YQ (2014). Interaction of fullerene chains and a lipid membrane via computer simulations. RSC Adv..

[44] Mhashal AR, Roy S (2014). Effect of gold nanoparticle on structure and fluidity of lipid membrane. PLoS One.

[45] Lin X, Li Y, Gu N (2010). Nanoparticle’s size effect on its translocation across a lipid bilayer: a molecular dynamics simulation. J. Comput. Theor. Nanosci..

[46] Marrink SJ, Tieleman DP (2013). Perspective on the Martini model. Chem. Soc. Rev..

[47] Hess B, Kutzner C, van der Spoel D, Lindahl E (2008). GROMACS 4: algorithms for highly efficient, load-balanced, and scalable molecular simulation. J. Chem. Theory Comput..

[48] Marrink SJ, Risselada HJ, Yefimov S, Tieleman DP, de Vries AH (2007). The MARTINI force field: coarse grained model for biomolecular simulations. J. Phys. Chem. B.

[49] Monticelli L (2012). On atomistic and coarse-grained models for C-60 fullerene. J. Chem. Theory Comput..

[50] Sridhar A, Srikanth B, Kumar A, Dasmahapatra AK (2015). Coarse-grain molecular dynamics study of fullerene transport across a cell membrane. J. Chem. Phys..

[51] Yang H, Huang Z, Zhang Y (2018). Effect of C60 on the phase transition behavior of a lipid bilayer under high pressure. Rsc Adv.

[52] Liang L, Kang Z, Shen JW (2016). Translocation mechanism of C60 and C60 derivations across a cell membrane. J Nanopart Res.

[53] Cherniavskyi YK, Ramseyer C, Yesylevskyy SO (2016). Interaction of C60 fullerenes with asymmetric and curved lipid membranes: a molecular dynamics study. Phys. Chem. Chem. Phys..

[54] Bussi G, Donadio D, Parrinello M (2007). Canonical sampling through velocity rescaling. J. Chem. Phys..

[55] Bussi G, Zykova-Timan T, Parrinello M (2009). Isothermal-isobaric molecular dynamics using stochastic velocity rescaling. J. Chem. Phys..

[56] Parrinello M, Rahman A (1981). Polymorphic transitions in single crystals: a new molecular dynamics method. J. Appl. Phys..

[57] Humphrey W, Dalke A, Schulten K (1996). VMD: visual molecular dynamics. J. Mol. Graph..

[58] Marsh D (1991). General features of phospholipid phase transitions. Chem. Phys. Lipids.

[59] Marrink SJ, Risselada J, Mark AE (2005). Simulation of gel phase formation and melting in lipid bilayers using a coarse grained model. Chem. Phys. Lipids.

[60] Lyubartsev AP, Rabinovich AL (2016). Force field development for lipid membrane simulations. Biochim. Biophys. Acta, Biomembr..

[61] Chen XJ, Tieleman DP, Liang Q (2018). Modulating interactions between ligand-coated nanoparticles and phase-separated lipid bilayers by varying the ligand density and the surface charge. Nanoscale.

[62] Xu Y (2017). Perturbation of the pulmonary surfactant monolayer by single-walled carbon nanotubes: a molecular dynamics study. Nanoscale.

[63] Chiu C-C (2010). Coarse-grained potential models for phenyl-based molecules: II. Application to fullerenes. J. Phys. Chem. B.

[64] Hsu P-C, Jefferies D, Khalid S (2016). Molecular dynamics simulations predict the pathways via which pristine fullerenes penetrate bacterial membranes. J. Phys. Chem. B.

[65] Javanainen M, Martinez-Seara H, Vattulainen I (2017). Excessive aggregation of membrane proteins in the Martini model. PLoS One.

[66] DeVane R (2010). Parametrization and application of a coarse grained force field for benzene/fullerene interactions with lipids. J. Phys. Chem. B.

[67] Daily MD, Olsen BN, Schlesinger PH, Ory DS, Baker NA (2014). Improved coarse-grained modeling of cholesterol-containing lipid bilayers. J. Chem. Theory Comput..

[68] Ding J, Ma E, Asta M, Ritchie RO (2015). Second-nearest-neighbor correlations from connection of atomic packing motifs in metallic glasses and liquids. Sci. Rep..

[69] Wong-ekkabut J, Karttunen M (2015). Molecular dynamics simulation of water permeation through Alpha hemolysin channel. J. Biol. Phys..

[70] Cao Z, Peng Y, Li S, Liu L, Yan T (2009). Molecular dynamics simulation of fullerene C60 in ethanol solution. J. Phys. Chem. C.

[71] Lyon DY, Adams LK, Falkner JC, Alvarez PJJ (2006). Antibacterial activity of fullerene water suspensions:  Effects of preparation method and particle size. Environ. Sci. Technol..

[72] MacCallum JL, Tieleman DP (2006). Computer simulation of the distribution of hexane in a lipid bilayer: spatially resolved free energy, entropy, and enthalpy profiles. J. Am. Chem. Soc..

[73] Chen KL, Bothun GD (2014). Nanoparticles meet cell membranes: probing nonspecific interactions using model membranes. Environ. Sci. Technol..

