# Supplementary material for: Dependence of fullerene aggregation on lipid saturation due to a balance between entropy and enthalpy
Source: Sci Rep. 2019 Jan 31;9:1037. doi: 10.1038/s41598-018-37659-4 (PMC6355782; doi:10.1038/s41598-018-37659-4)
Supplement: Supplementary file 1 — Supporting Information [file 41598_2018_37659_MOESM1_ESM.docx]

**Supplementary Information**

**Dependence of fullerene aggregation on lipid saturation due to a balance between entropy and enthalpy**

Pornkamon Nalakarn^a,b,c,†^, Phansiri Boonnoy^a,b,†^, Nililla Nisoh ^a,b,c^, Mikko Karttunen^*,d^ and Jirasak Wong-ekkabut^*, a,b,c,e^

^a^ Department of Physics, Faculty of Science, Kasetsart University, Bangkok 10900, Thailand

^b^ Computational Biomodelling Laboratory for Agricultural Science and Technology (CBLAST), Faculty of Science, Kasetsart University, Bangkok 10900, Thailand

^c^ Thailand Center of Excellence in Physics (ThEP Center), Commission on Higher Education, Bangkok 10400, Thailand

^d^ Department of Chemistry and Department of Applied Mathematics, Western University, 1151 Richmond Street, London, Ontario N6A 5B7, Canada

^e^ Specialized Center of Rubber and Polymer Materials for agriculture and industry (RPM), Faculty of Science, Kasetsart University, Bangkok 10900, Thailand

†These authors contributed equally to this work

*Corresponding E-mail: jirasak.w@ku.ac.th and mkarttu@uwo.ca

**Figures**

| **Figure S1.** The coarse-grained (CG) structures of 1,2-dipalmitoyl-*sn*-glycero-3-phosphocholine (DPPC), 1,2-dioleoyl-*sn*-glycero-3-phosphocholine (DOPC), and 1-palmitoyl-2-oleoyl-*sn*-glycero-3-phosphocholine (POPC) molecule with the names of the CG beads in the Martini model^1^ and the CG fullerene molecule(F16)^2^. | 3 |
| --- | --- |
| **Figure S2.** Top and side views of the final structures at 20 microseconds for the systems at concentrations of 0% and 5% fullerene in DPPC, DOPC, and POPC lipid bilayers. | 4 |
| **Figure S3.** Mass density profiles of the lipid components and fullerene along the bilayer normal (z-axis) for the system with 5%,40%, and 50% of fullerene in DPPC and DOPC bilayers. | 5 |
| **Figure S4.** Mass density profiles of the lipid components and fullerene along the bilayer normal (*z*-axis) in the POPC bilayer with different concentrations of fullerene. | 6 |
| **Figure S5**. Area compressibility (κ_A_) of different lipid bilayers.  **Figure S6.** Time evolution of fullerene aggregation and entry into the DPPC lipid bilayer at concentrations of 20% and 40%. The red dashed vertical lines show when the last fullerene entered the membrane. | 7  8 |
| **Figure S7.** The fraction percentage of fullerenes in the largest cluster as a function of fullerene concentration in the DPPC, DOPC, and POPC bilayers. | 9 |
| **Figure S8**. Free energy profiles of fullerenes in lipid bilayers at different concentrations. | 10 |
| **Figure S9.** Free energy profiles of fullerenes in DPPC bilayers with 512 and 2048 lipids at concentrations of 10% and 30%. | 11 |


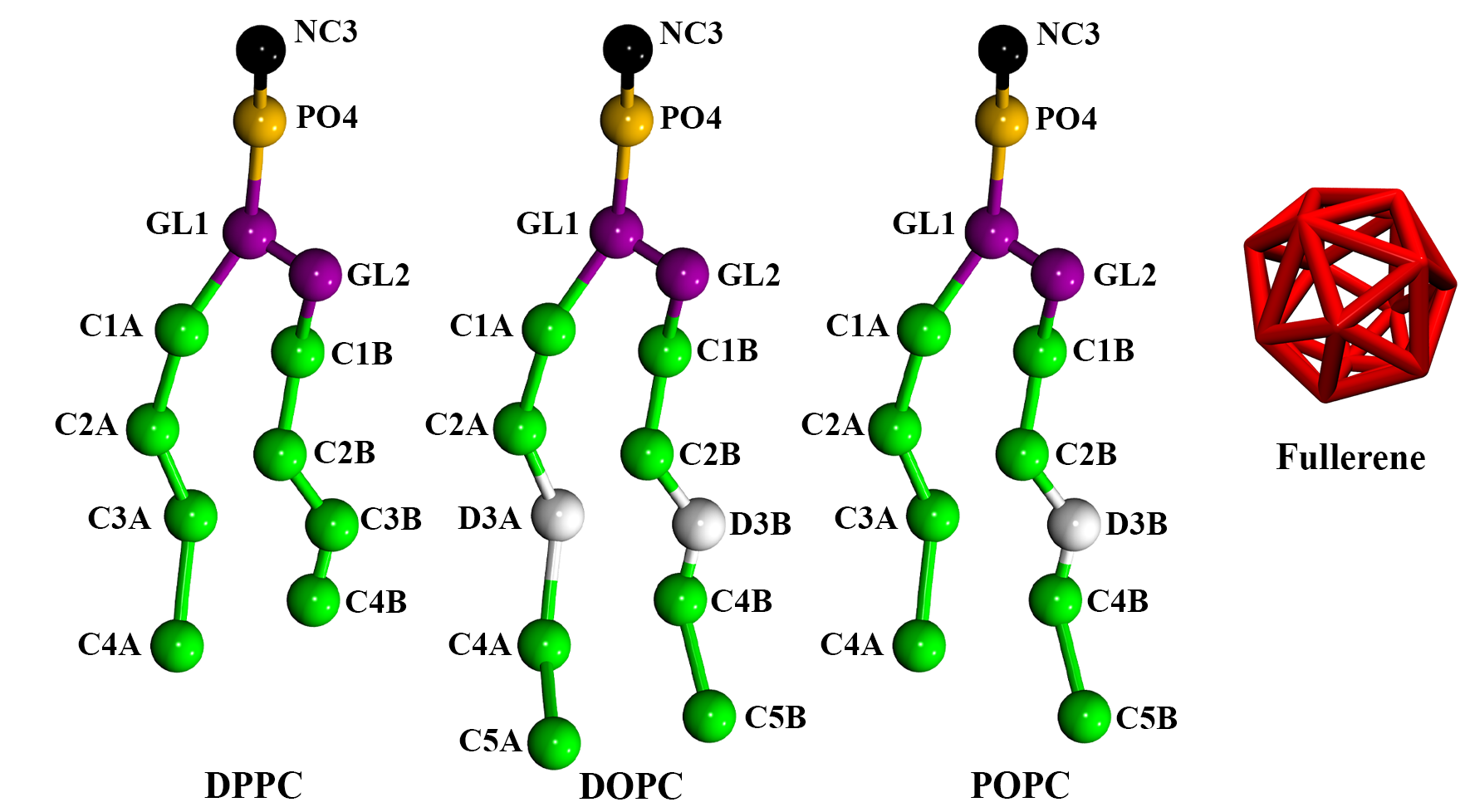


**Figure S1.** The coarse-grained (CG) structures of 1,2-dipalmitoyl-*sn*-glycero-3-phosphocholine (DPPC), 1,2-dioleoyl-*sn*-glycero-3-phosphocholine (DOPC), and 1-palmitoyl-2-oleoyl-*sn*-glycero-3-phosphocholine (POPC) molecule with the names of the CG beads in the Martini model^1^ and the CG fullerene molecule (F16)^2^.


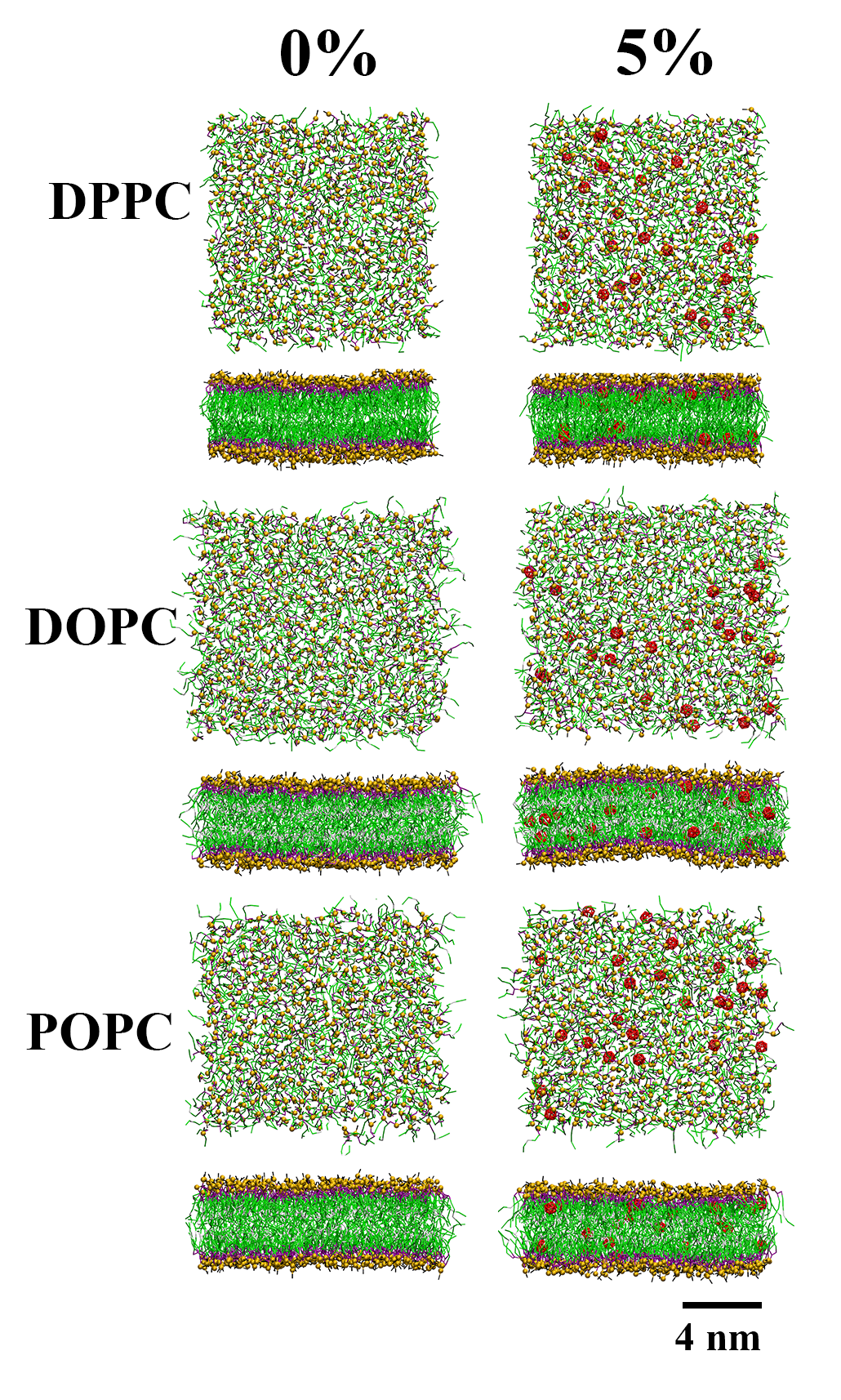


**Figure S2.** Top and side views of the final structures after 20 microseconds for the system at 0% and 5% of fullerene in DPPC, DOPC, and POPC lipid bilayers. The other systems are shown in the main text.


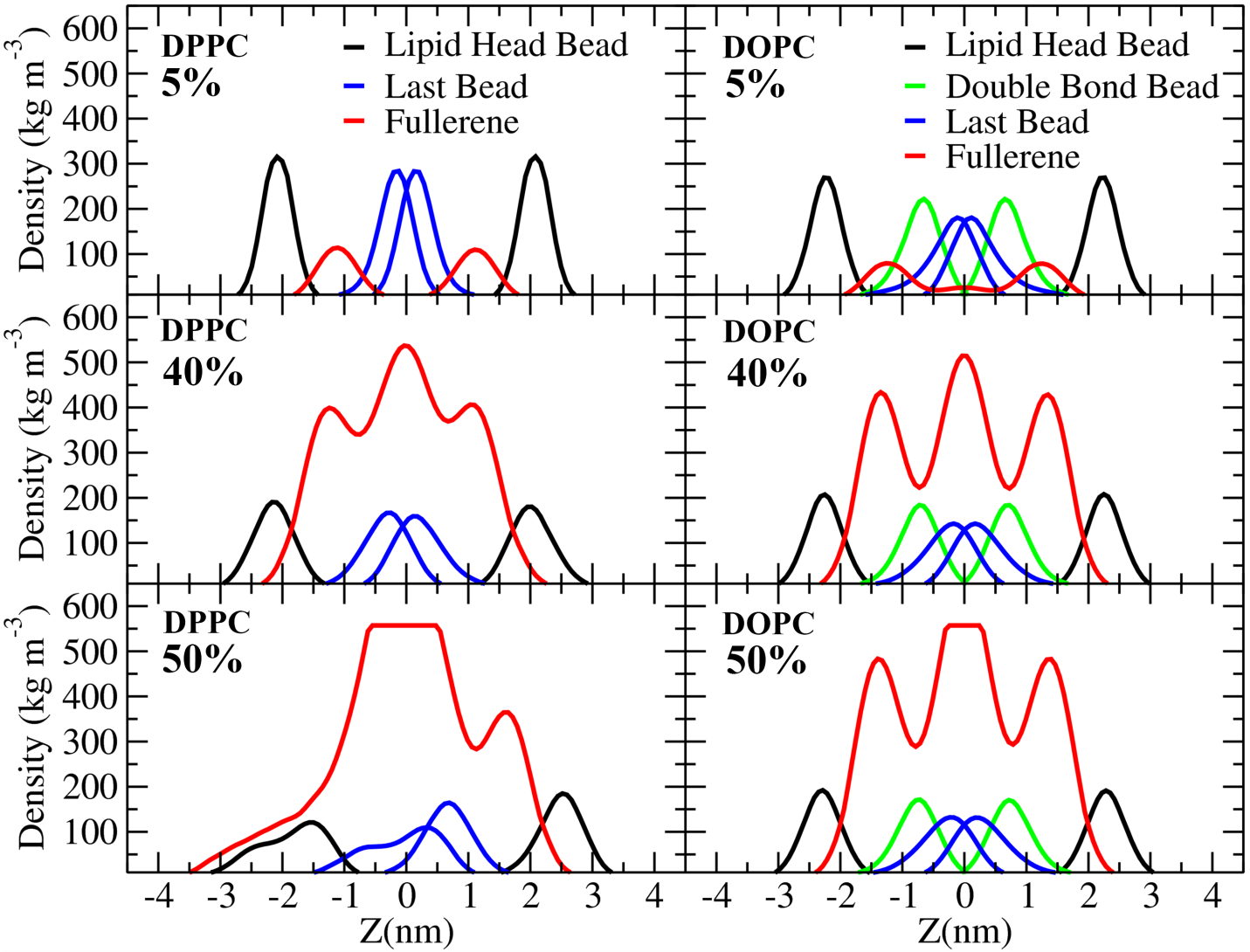


**Figure S3.** Mass density profiles of the lipid components and fullerene along the bilayer normal (z-axis) for the system with 5%,40%, and 50% of fullerene in DPPC and DOPC bilayers. Note that lipid head bead, double bond bead and the last bead are PO4, D3B and C4B(DPPC)/C5B(DOPC), repectively.


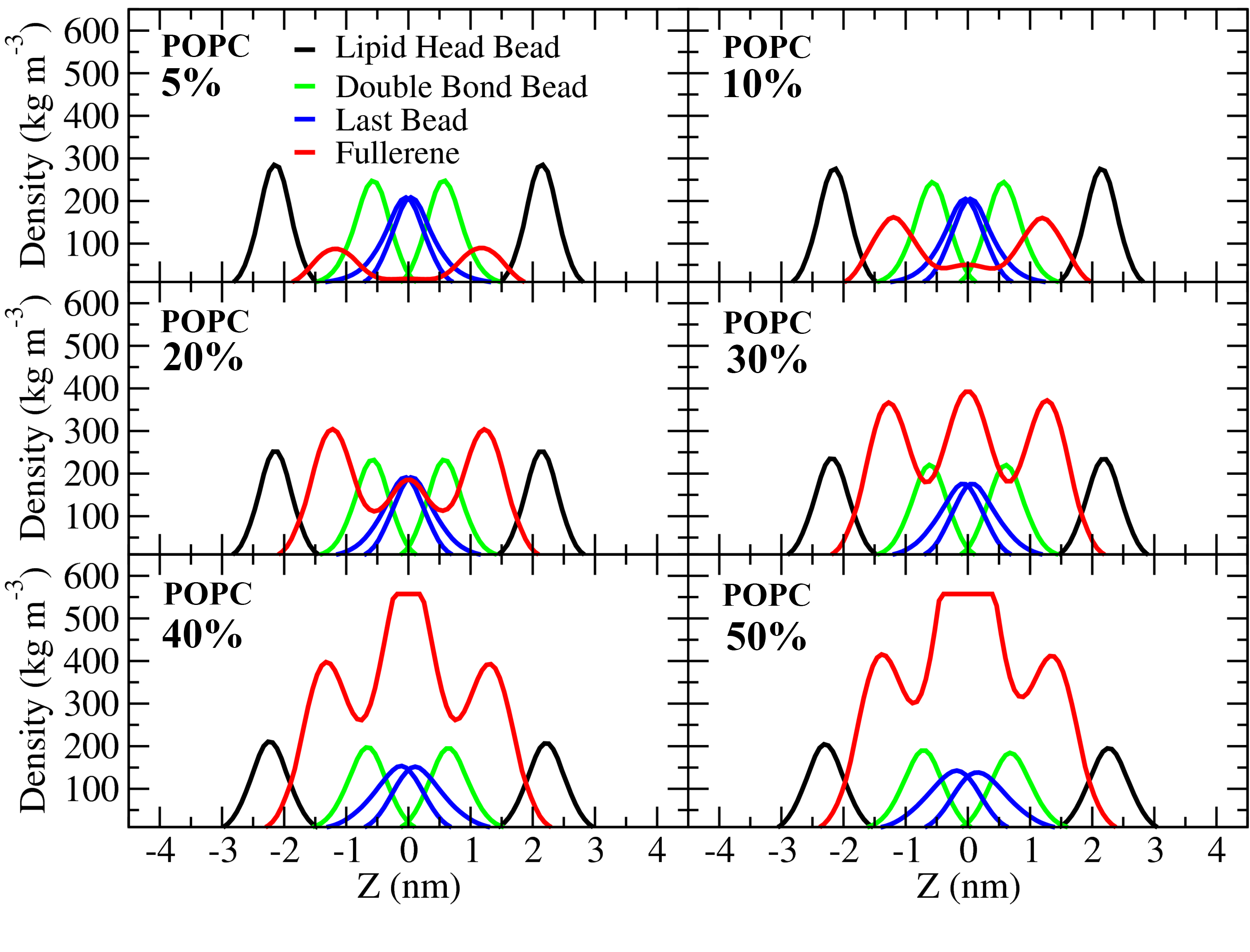


**Figure S4**. Mass density profiles of the lipid components and fullerene along the bilayer normal (z-axis) in the POPC bilayer with different concentrations of fullerene. Note that lipid head bead, double bond bead and the last bead are PO4, D3B and C5B, repectively.


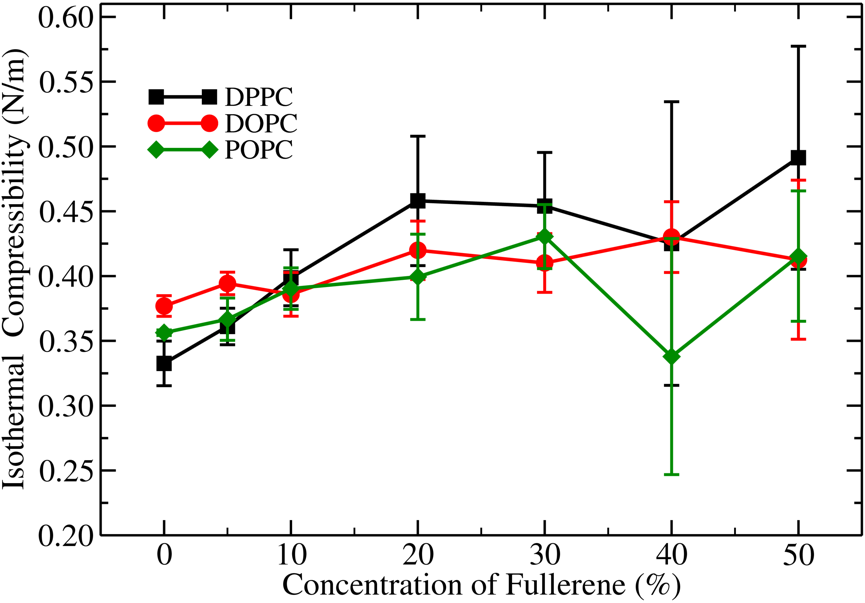


**Figure S5**. Area compressibility (κ_A_) of different lipid bilayers.


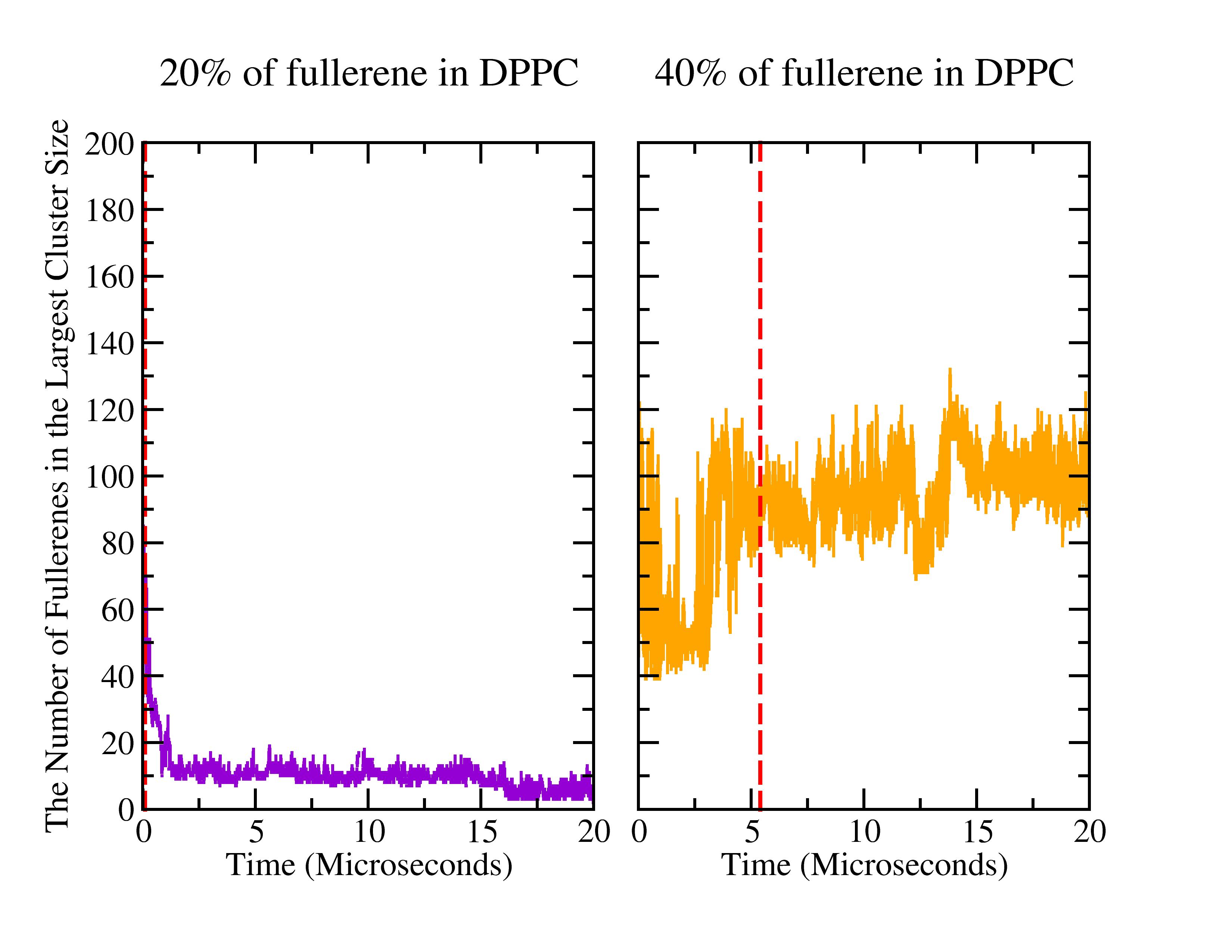


**Figure S6.** Time evolution of fullerene aggregation and entry into the DPPC lipid bilayer at concentrations of 20% and 40%. The red dashed vertical line shows when the last fullerene entered the membrane.


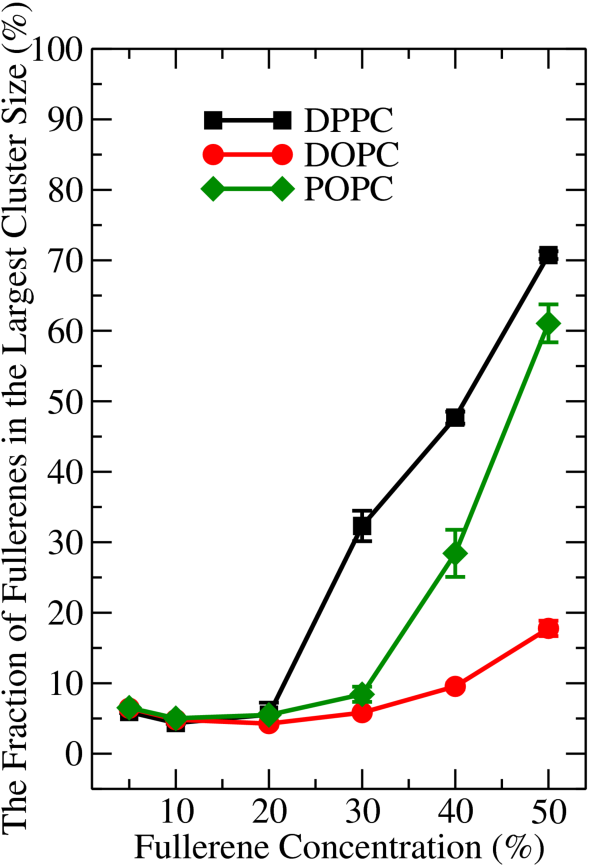


**Figure S7**. The fraction percentage of fullerenes in the largest cluster as a function of the concentration of fullerene in the DPPC, DOPC, and POPC bilayers.


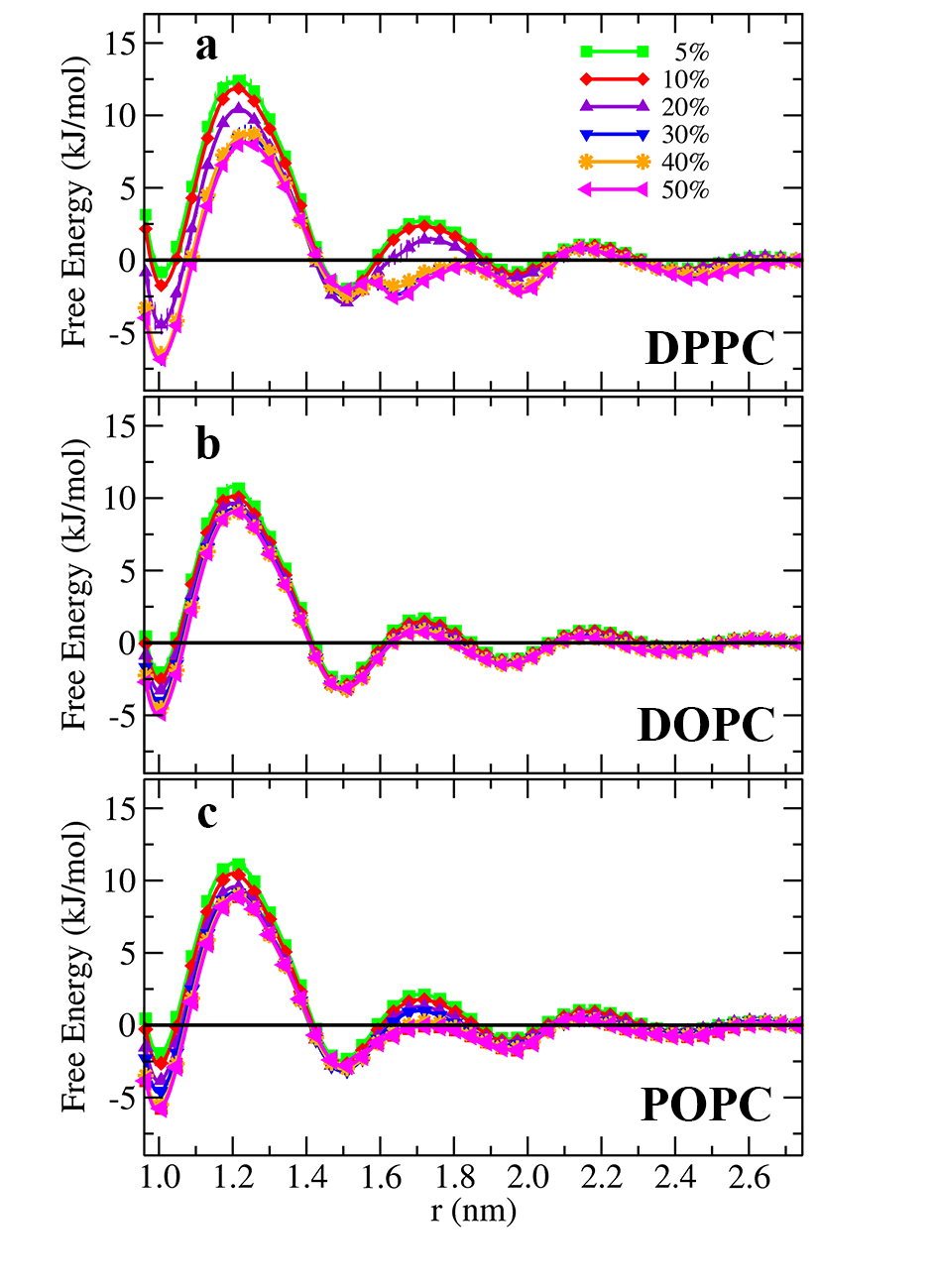


**Figure S8**. Free energy profiles of fullerenes in lipid bilayers at different concentrations.


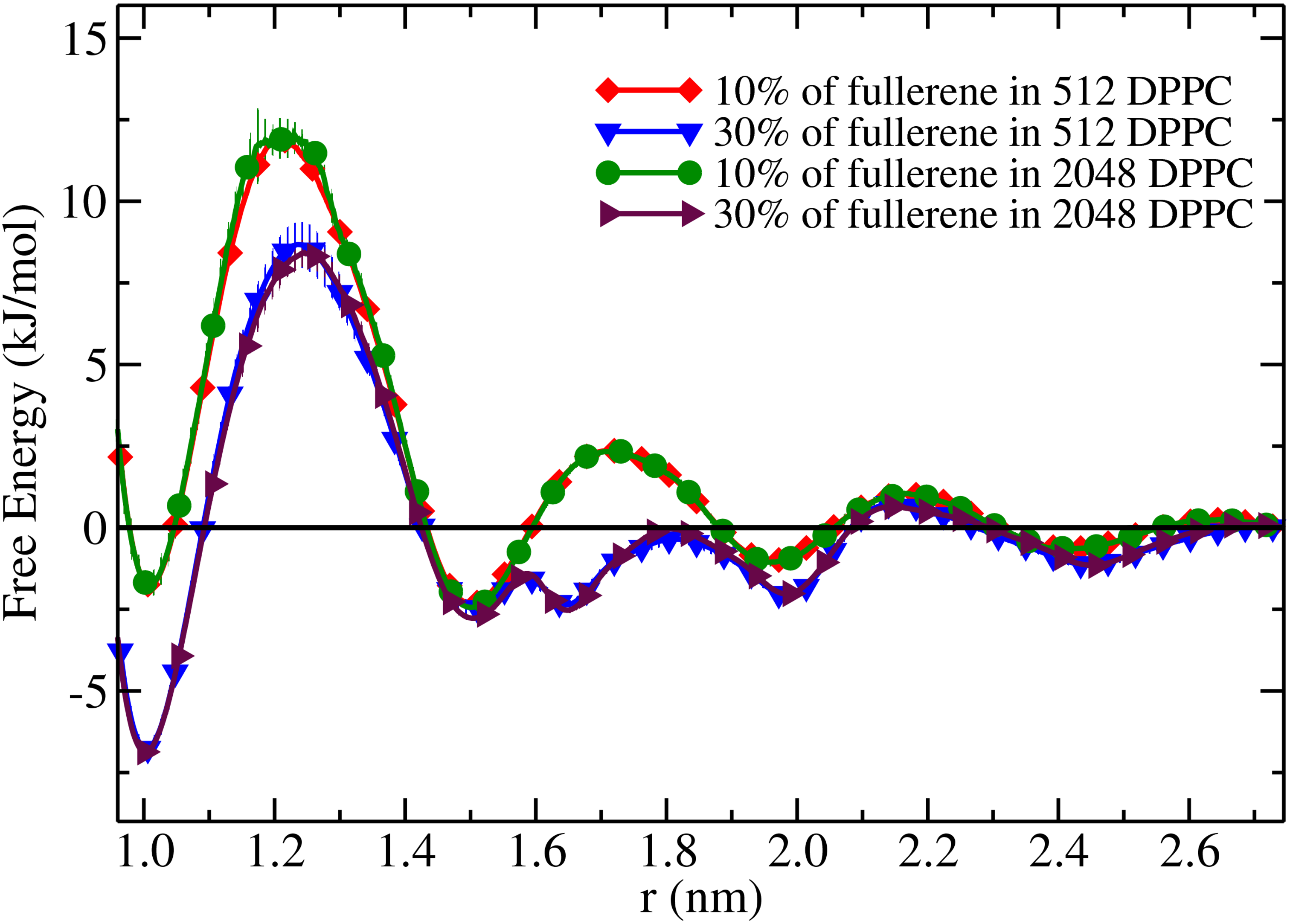


**Figure S9.** Free energy profiles of fullerenes in DPPC bilayers having 512 and 2048 lipids at concentrations of 10% and 30%.

**References**

1 Marrink, S. J., Risselada, H. J., Yefimov, S., Tieleman, D. P. & de Vries, A. H. The MARTINI force field: Coarse grained model for biomolecular simulations. *J. Phys. Chem. B* **111**, 7812-7824 (2007).

2 Monticelli, L. On atomistic and coarse-grained models for C-60 fullerene. *J. Chem. Theory Comput*. **8**, 1370-1378 (2012).
